# Prevalence of Individuals With Multiple Diagnosed Genetic Diseases in the Undiagnosed Diseases Network

**DOI:** 10.1002/ajmg.a.63888

**Published:** 2024-09-27

**Authors:** Alex F. Gimeno, Rory J. Tinker, Yutaka Furuta, Undiagnosed Diseases Network, John A. Phillips III

**Affiliations:** Division of Medical Genetics and Genomic Medicine, Vanderbilt University Medical Center, Nashville, Tennessee, USA

**Keywords:** multiple genetic diseases, rare genetic disease, Undiagnosed Diseases Network

## Abstract

Undiagnosed Diseases Network Report the prevalence of multiple genetic diseases in the Undiagnosed Diseases Network (UDN) cohort in the post-exome-sequencing era. UDN subjects underwent genome sequencing before inclusion in the cohort. Records of all UDN subjects until January 2024 were analyzed. The number of diagnoses, proportion of molecular versus nonmolecular (i.e., not attributable to a discretely identifiable genetic change) diagnoses, and the inheritance patterns of the genetic diagnoses were determined. Of 2799 subjects, 766 (27.4%) had diagnoses. Of these 766, 95.4% had one diagnosis, 4.0% had two diagnoses, and 0.5% had three diagnoses. Of the diagnosed subjects, 93.4% had a genetic disease, and 6.5% had a nonmolecular disease. Of subjects with two diagnoses, both diagnoses were molecular in 90.3%, while 9.7% had one molecular and one nonmolecular diagnosis. All four subjects with three diagnoses had three molecular diagnoses. 4.2% of diagnosed subjects in the UDN had more than one molecular diagnosis, with four individuals having three concurrent Mendelian diagnoses. Additionally, three subjects had concurrent molecular and nonmolecular diagnoses. Given that numerous UDN subjects had a negative genome sequence prior to UDN enrollment, multiple molecular diagnoses may contribute to diagnostic uncertainty even with genome sequencing, as may concurrent nonmolecular disease.

## 1 | Introduction

Per the Orphan Drug Act, a rare disease is defined in the United States as any disease affecting < 200,000 people in the country (Herder 2017) and therefore reflected by a prevalence of ~1 in 1900 in the population. Of these rare diseases, 80% are thought to be genetic in origin (Frederiksen et al. 2022). While any given rare disease is individually uncommon, estimates put the total population burden of rare diseases at around 2% of the population. The presence of a rare disease is associated with increased rates of hospitalization for patients as well as increased healthcare expenditures for their families (Ferreira 2019). After diagnosis of a rare disease, healthcare costs, hospitalizations, and procedures are reported to drop precipitously; however, diagnosis is often delayed on the order of years to decades (Tinker, Fisher et al. 2024). Given this, improved detection and diagnosis of rare disease presents an opportunity to improve patient well-being and the cost-effectiveness of the healthcare system (Chung et al. 2020).

While the importance of biotechnology and molecular diagnostic techniques have increased for diagnosing unknown diseases, patient’s medical histories and physical examinations as well as health care providers’ clinical reasoning remain essential to guide the choice of tests given the large number of available test options. Patients presenting more than one, or multiple genetic diseases (MGD) can make diagnoses especially difficult. Examples include dual diagnoses of maternal 10q11.22q11.23 microduplication syndrome presenting together with *WDR19*-related recessive ciliopathy, Down syndrome presenting together with *LAMA2*-associated muscular disease, and 16p11.2 microdeletion presenting together with Stargardt disease, as reported in (Capra et al. 2023). Previously, exome sequencing (ES) studies of patients with geneticist-suspected disease showed that 3.5% of these patients had MGD (Balci et al. 2017). Other studies report similar diagnostic yields, with the proportion of patients with MGD found by ES ranging from 0.4% to 4%, with the range increasing to 1.4%–8.5% when only solved cases were considered (Farwell et al. 2015; Lee et al. 2014; Posey et al. 2017; Retterer et al. 2016; Yavarna et al. 2015). Thus, the presence of MGD is a well-documented phenomenon that is worth investigating, as diagnosis may be delayed if a single disease is thought to explain all of a patient’s symptoms when MGD should be higher on a clinician’s differential diagnosis.

The Undiagnosed Diseases Network (UDN) has played an important role in solving elusive diagnoses in the United States. By combining the efforts of expert clinicians and research-grade next-generation sequencing (both genomic and exome), the UDN strives to improve the levels of diagnosis and care, facilitate research on undiagnosed diseases, and create a collaborative research community to identify improved options for patient care. The UDN has already helped advance understanding in such topics as the prevalence of mosaicism in undiagnosed diseases (Tinker et al. 2023) and the role of model organism work in genetic research (Baldridge et al. 2021). However, the UDN data have yet to be examined with regards to MGD, despite the substantial impact these diseases can have on study subjects.

The goal of the current study was therefore to establish the proportion of UDN subjects with MGD for the first time. These results will help clinicians better adjust their indices of suspicion for MGD in diagnostically challenging cases and hopefully allow for quicker diagnoses of patients with MGD.

## 2 | Materials and Methods

### 2.1 | Editorial Policies and Ethical Considerations

This study was conducted under UDN Institutional Review Board number: 15HG0130.

### 2.2 | Sample Composition

All subject data were drawn from the shared UDN database. The UDN was founded by the National Institutes of Health in 2014 and has evolved into a collaborative network made up of researchers and clinicians from 12 clinical sites, a coordinating center, a biorepository, and various core laboratories that provide resources for sequencing, model organism work, and metabolomics. Subjects were not required to have genome sequencing prior to enrollment.

### 2.3 | Definitions for Analysis

A UDN subject who had received any diagnosis included any subject that had a diagnosis that partially or completely explained their phenotype. This included subjects who had a diagnosis prior to inclusion in the UDN cohort and whose UDN workup did not demonstrate the presence of a second genetic disease. Prior to UDN testing, a UDN provider reviewed the referring note and all other available medical information (including laboratory testing, family history, and pertinent images) and then generated a potential list of human phenotype ontology (HPO) terms and differential diagnoses (Splinter et al. 2018). More specifically, UDN diagnoses were made with a multidisciplinary committee-based approach based on group consensus of experts in clinical and laboratory genetics. For particularly challenging cases, less common techniques such as animal modeling and metabolomics were occasionally used. Variant interpretation was performed based on consensus guidelines as applied by the ACMG (Richards et al. 2015; Riggs et al. 2020; Splinter et al. 2018) and included both known disease genes/variants as well as candidate variants/genes. When variants were reclassified to causative from uncertain, this was done based on group consensus using supportive studies as outlined below. As UDN clinicians consider all diseases, and not just genetic disorders, nonmolecular diagnoses were determined in consultation with a disease-specific expert after a putative nonmolecular diagnosis (which would partially or completely explain the subject’s phenotype) had been proposed (Splinter et al. 2018). The method of diagnosis was recorded by clinicians at each UDN site.

Molecular diagnoses included any diagnosis due to discretely identifiable molecular changes, including Mendelian changes, mosaicism, digenic changes, cytogenetic changes, uniparental disomy, imprinting defects, and mitochondrial disease. A distinction between Mendelian and non-Mendelian diagnoses was made to aid comparability between studies, as some prior studies report rates of Mendelian disease only. Mendelian diseases were counted as those diseases that traditionally follow a simple autosomal dominant, autosomal recessive, or X-linked dominant or recessive inheritance pattern. Non-Mendelian molecular diagnoses referred to mosaicism, digenic changes, cytogenetic changes, uniparental disomy, imprinting defects, and mitochondrial disease. Cytogenetic disease referred to partial or whole chromosomal duplications, deletions, and translocations (vs. changes at a single allele). In the context of the current study, MGD was taken to mean the presence of more than one molecular diagnosis in a subject. Finally, non-molecular diagnoses were defined as those diagnoses that at least partially explained subject symptoms (as determined by clinicians specialized in the diagnosis in question) but are not inherited or caused via traditional Mendelian, mosaic, mitochondrial, cytogenetic, or digenic means. In the current study, nonmolecular diagnoses were those diagnoses due to the combined effects of multiple genetic variants and environmental risk factors, including autoimmune diseases and autism not due to a single gene variant. This terminology was chosen to distinguish conditions with discretely identifiable genetic bases (which may be more amenable to genetic testing) from those that contain many small genetic as well as environmental contributions (for which genetic testing is less helpful). Because the term molecular has historically been used to refer to diseases due to discrete problems with a given enzyme or protein (Cammack et al. 2008), the authors chose this term to refer to Mendelian conditions, mosaicism, digenic conditions, cytogenetic conditions, uniparental disomy-related conditions, imprinting defects, and mitochondrial disease. The authors acknowledge that there are multiple definitions of molecular diagnosis, and that some include only monogenic changes; however, given that one of the goals of the current study was to investigate the rate of genetic diseases with discretely identifiable etiologies that are more amenable to genetic testing versus those diagnoses containing multiple smaller genetic contributions for which genetic testing may be less helpful, the authors decided that use of the current terminology would suffice. Analyses of Mendelian (i.e., monogenic) versus non-Mendelian disease rates have also been included for readers wishing to take a stricter definition of molecular disease.

### 2.4 | Tabulation of Results

As of 29 January 2024, the UDN had completed evaluation of 2799 subjects and had diagnosed 766 of them, yielding a final sample size of 766 subjects with a known diagnosis (genetic or otherwise) and a diagnostic rate of 766/2799 (27.4%). A subject was counted as successfully diagnosed when a DNA variant was found by the UDN that was consistent with and could explain part or all of the subject’s presenting phenotype, or when genetic testing ruled out genetic causes for symptoms and a different, nonmolecular diagnosis was found and confirmed.

Once the sample of diagnosed subjects was determined, subjects were stratified into groups based on number of diagnoses (that is, if the subject had one, two, or three diagnoses). Next, the specific diagnosed condition or conditions for each subject (as well as the associated gene, if applicable) was examined to calculate the proportion of subjects in each group with diseases associated with a single genetic disease.

### 2.5 | Diagnostic Delay Analysis

Given the increasing interest in diagnostic delay in the genetics literature (Tinker, Fisher et al. 2024), age at presentation and diagnostic delay were also analyzed for subgroups of interest. For these analyses, diagnostic delay was defined as the difference between date of diagnosis and date of symptom onset; for those subjects with multiple diagnoses, the longest diagnostic delay was used in analysis. First, diagnostic delay was compared between subjects with one molecular diagnosis and one nonmolecular diagnosis using a one-tailed independent-samples *t*-test (as subjects with nonmolecular diagnoses were anticipated to have longer diagnostic odysseys). Next, diagnostic delay was compared pairwise between subjects with one, two, and three diagnoses using one-tailed independent-samples *t*-tests (as subjects with more diagnoses were anticipated to have longer diagnostic delays). Then, diagnostic delay was compared across general diagnostic methods (i.e., genome-scale sequencing, directed clinical testing, etc.) for all subjects with diagnostic delay data and one diagnosis using a one-way ANOVA.

Age at UDN presentation was also compared between subjects with one molecular versus one nonmolecular diagnosis using a one-tailed independent-samples *t*-test (as it was predicted that nonmolecular diagnoses would have a later age at presentation than molecular diagnoses). Age at UDN presentation was also compared between groups with one, two, and three diagnoses one-tailed independent-samples *t*-test (as it was predicted that age at presentation would increase with number of diagnoses).

## 3 | Results

### 3.1 | UDN Sample Characteristics

The UDN cohort initially consisted of 2799 subjects with completed UDN evaluations recruited from August 2017 through January 2024. Of these, 766/2799 (27.4%) received a diagnosis and were included for analysis in our study. The final sample contained 388 female subjects (50.7%) and 377 male subjects (49.2%). The racial breakdown was 79.4% White, 8.0% Asian, 5.1% Black/African American, 4.7% mixed race, 1.4% unspecified, 0.8% other, 0.5% American Indian or Alaskan native, and 0.1% Native Hawaiian or other Pacific islander (Table S1). Briefly, 127 (16.6%) of subjects identified as Hispanic/Latino, 574 (74.9%) identified as not Hispanic or Latino, and 65 (8.5%) did not report this information.

Of the 766 subjects analyzed, 95.4% (731/766 subjects) had only one diagnosis, 4.0% (31/766 subjects) had two diagnoses, and 0.5% (4/766 subjects) had three diagnoses; therefore, of the diagnosed UDN cohort, 35 subjects (4.6%) had multiple diagnoses, 93.9% (719/766 subjects) had at least one molecular diagnosis. No subjects had more than three diagnoses. For the 35 cases with more than one diagnosis, 20 (57%) had at least ES prior to UDN enrollment. Of these 20, only 2 (10%) had a variant identified on initial ES that ultimately corresponded to the final diagnosis. When exome sequences were re-analyzed, variants were identified for 19 subjects.

**FIGURE 1 |:**
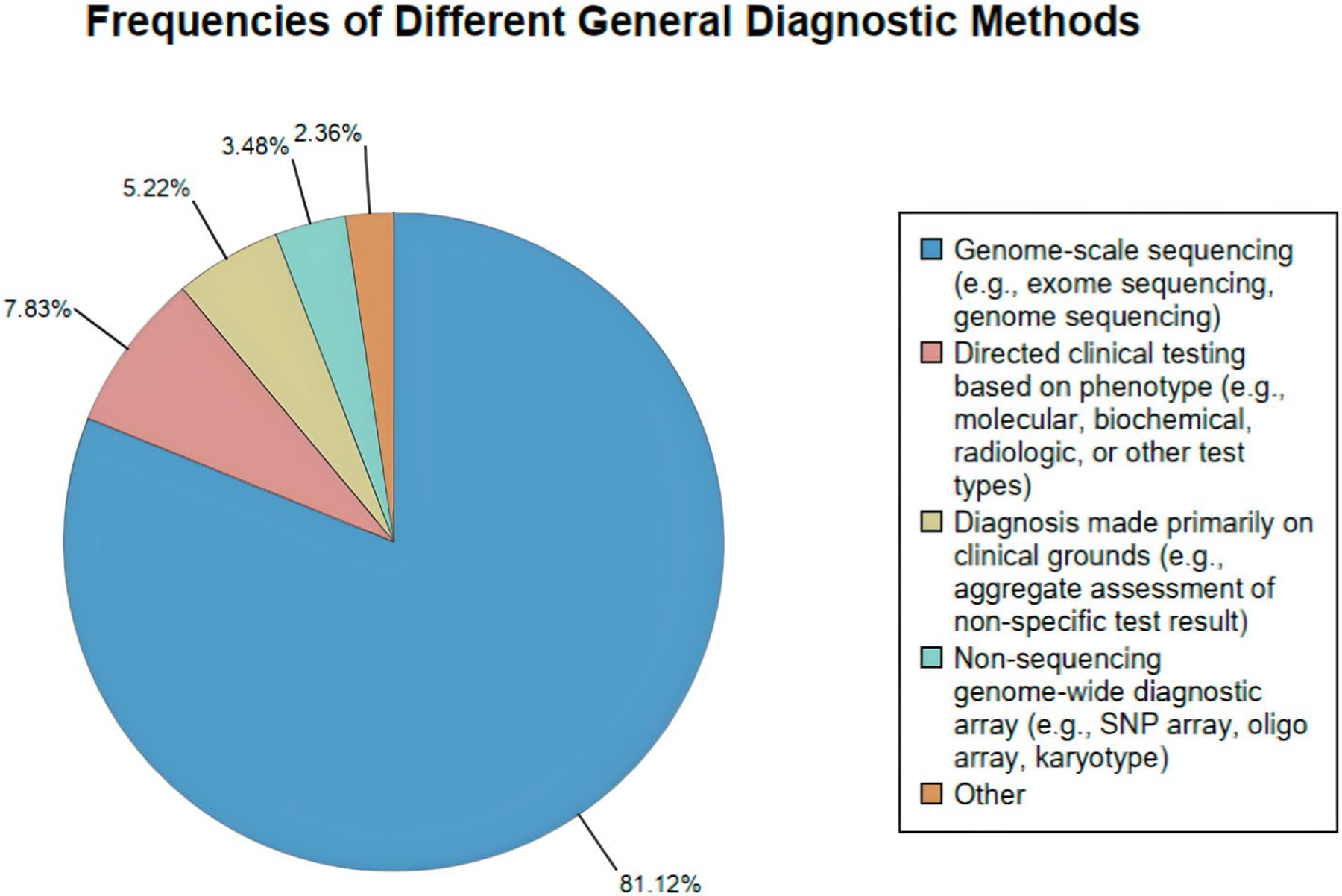
Frequencies of different general diagnostic methods of diagnosed UDN participants.

Therefore, a total of 805 diagnoses were made. Of these, 653 (81.1%) were made using genome-scale sequencing, 63 (7.8%) were made using directed clinical testing (including imaging and laboratory testing), 42 (5.2%) were made primarily on clinical grounds, 28 (3.5%) were made based on non-sequencing genome-wide testing, and 19 (2.4%) were made with other methods (Figure 1 and Tables S3–S7). With regards to the 62 diagnoses for the 31 subjects with 2 diagnoses, 48 (77.4%) were made using genome-scale sequencing, 3 (4.8%) were made using directed clinical testing, 2 (3.2%) were made primarily on clinical grounds, 6 (9.7%) were made based on non-sequencing genome-wide testing, and 3 (4.8%) were made using another method. For the 12 diagnoses from the 4 subjects with 3 diagnoses, 11 diagnoses were made using genome-scale-sequencing, and 1 with directed clinical testing based on phenotype.

**FIGURE 2 |:**
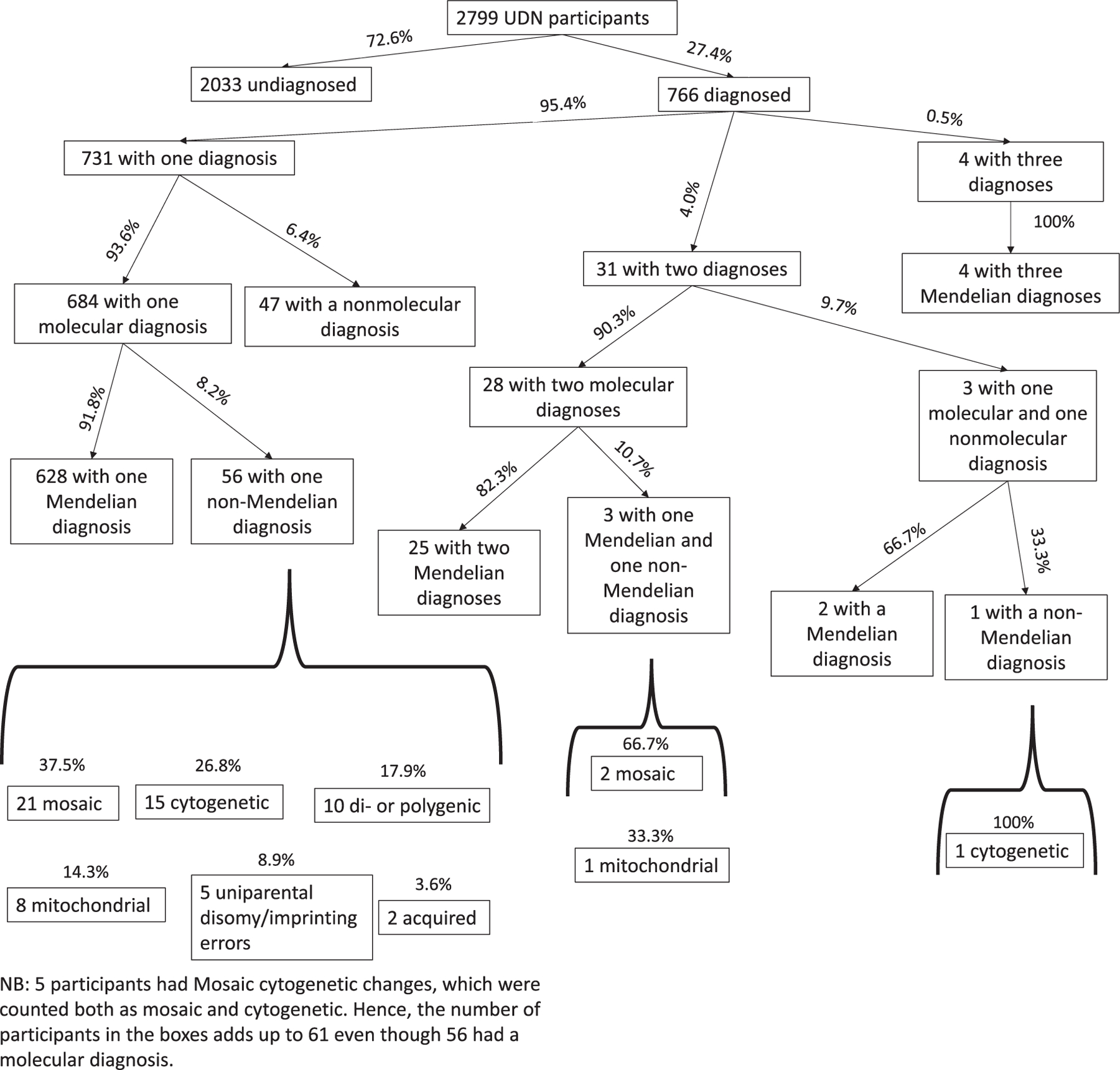
Characteristics of the Undiagnosed Diseases Network (UDN) cohort analyzed in the current study.

Of the 766 diagnosed subjects, 32 (4.2%) had multiple molecular diagnoses only (that is, two or three molecular diagnoses, Mendelian or non-Mendelian, without any nonmolecular comorbidities). Of the 35 subjects with multiple molecular diagnoses, 29 had only Mendelian genetic diseases (82.3%) (Figure 2). The subjects with multiple molecular diagnoses including one that was non-Mendelian were: van Maldergem Syndrome 2 and diploid–triploid mosaicism (mosaic); intellectual developmental disorder, autosomal dominant 42 and myoclonic epilepsy with ragged red fibers (mitochondrial), and NRCAM-related neurodevelopmental disorder and *KRAS*-related RASopathy (mosaic).

### 3.2 | Mendelian Disease Characteristics

In the cohort of 766 diagnosed subjects, 662 (86.4%) had at least one Mendelian disease; this was 662/2799 (23.7%) of the entire UDN cohort. Of the 731 subjects with one diagnosis, 628 (85.9%) had a diagnosis that was a Mendelian disease (i.e., with simple autosomal dominant/recessive or X-linked inheritance); the other 103 subjects had diseases that were of non-Mendelian inheritance (mosaic, cytogenic, di-/polygenic, mitochondrial, due to uniparental disomy/imprinting errors, or acquired; 56 subjects out of 731 [7.7%]) or that were nonmolecular altogether (47 subjects out of 731 [6.4%]). Of the 766 diagnosed UDN subjects, 25 (3.3%) had two Mendelian diagnoses and 4 (0.5%) had three Mendelian diagnoses (Figure 2).

### 3.3 | Molecular and Nonmolecular Disease Characteristics

Of the 766 diagnosed subjects, 719 (93.9%) had at least one molecular diagnosis and 50 (7.0%) had at least one nonmolecular diagnosis; 3 subjects (0.4%) had both molecular and nonmolecular diagnoses. Of the 766 subjects with a diagnosis, 662 (86.4%) had at least one molecular disease with Mendelian inheritance, while 60 (7.8%) had at least one molecular disease with non-Mendelian inheritance.

**FIGURE 3 |:**
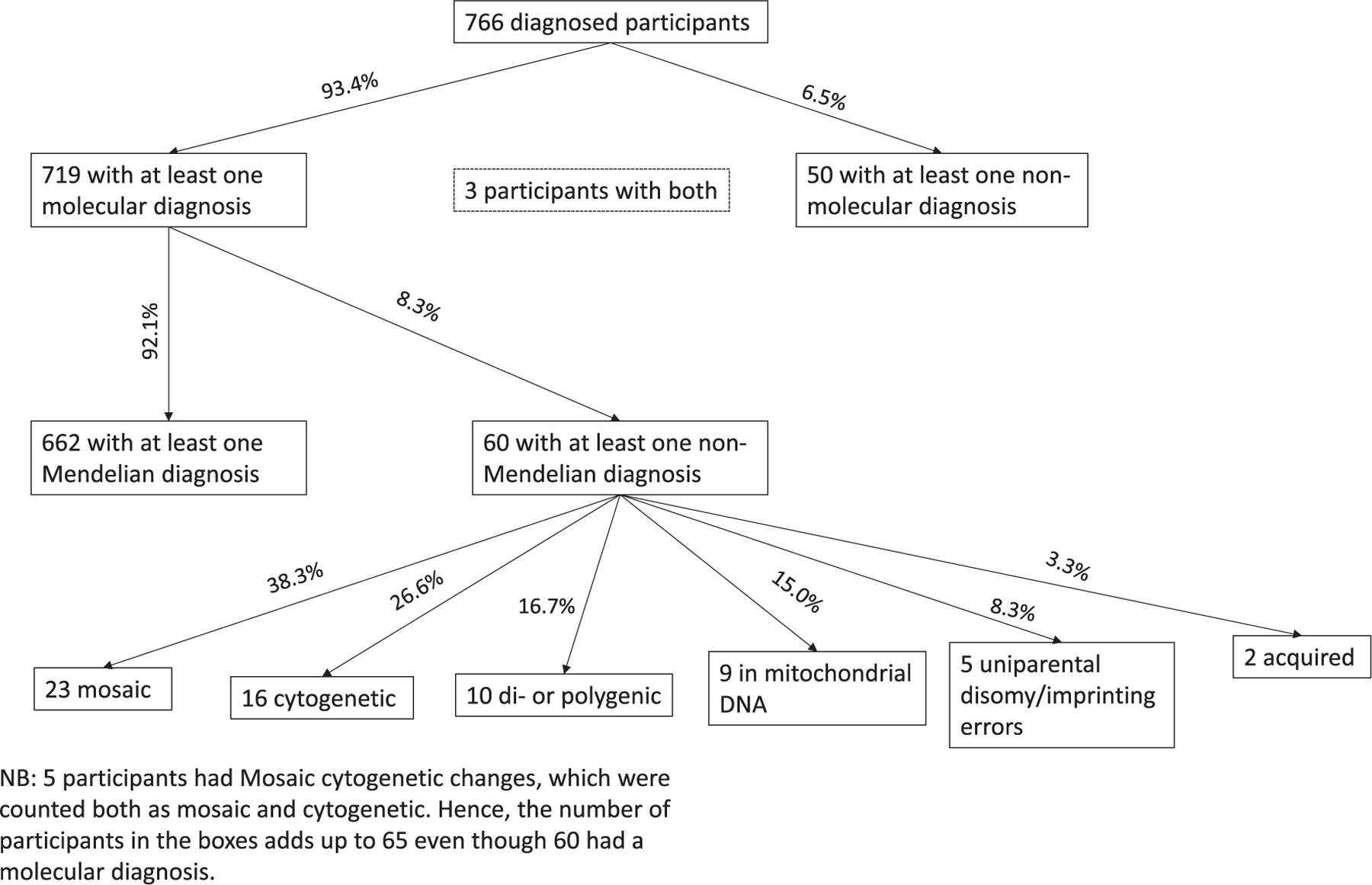
Characteristics of diagnoses in the Undiagnosed Diseases Network (UDN) cohort analyzed in the current study.

Of the 60 cases with non-Mendelian inheritance, there were 23 subjects with mosaic conditions (38.3%), 16 subjects with cytogenetic conditions (26.7%), 10 subjects with di- or polygenic conditions (22.2%), 9 subjects with mitochondrial DNA mutations or deletions (15.0%), 5 subjects with conditions due to uniparental disomy or imprinting errors (8.3%), and 2 subjects with conditions due to an acquired genetic change (i.e., VEXAS syndrome) (3.3%) (Figure 3).

Of the 35 subjects with multiple (genetic and nonmolecular) diagnoses, 6/35 (17.1%) had at least one non-Mendelian disease (either molecular non-Mendelian or nonmolecular). These included 2 subjects with multiple sclerosis, 1 subject with systemic lupus erythematosus, 1 subject with diploid-triploid mosaicism, 1 subject with mosaic *KRAS*-related RASopathy, and 1 subject with Myoclonic epilepsy with ragged-red fibers (MERRF) (Table 1). All 4 subjects with 3 diagnoses had 3 Mendelian diseases (Table 2). Therefore, of the diagnosed cohort, the number of subjects with exclusively Mendelian MGD was 29/766 (3.8%), and the number of subjects with any MGD was 32/766 (4.2%).

### 3.4 | Diagnostic Delay and Age at Presentation Analyses

Regarding diagnostic delay in subjects with one diagnosis, the 580 subjects with one molecular diagnosis (mean = 10.8 ± 9.4 years), compared to the 45 subjects with one nonmolecular diagnosis (mean = 9.5 ± 7.2 years), did not have significantly different diagnostic delays (*t*(56.2) = 1.12, *p* = 0.13). Equal variances were not assumed for this test based on Levene’s test for equality of variances.

Regarding diagnostic delay by number of diagnoses, the 625 subjects with one diagnosis had a mean diagnostic delay of 10.7 ± 9.2 years, the 27 subjects with two diagnoses had a mean diagnostic delay of 10.0 ± 5.8 years, and the 4 subjects with three diagnoses had a mean diagnostic delay of 12.5 ± 9.9 years. There was no statistical difference in diagnostic delay between subjects with and two diagnoses (*t*(650) = 0.36, *p* = 0.36), one and three diagnoses (*t*(3.0) = −0.37, *p* = 0.37), and two and three diagnoses (*t*(29) = −0.73, *p* = 0.24).

Regarding diagnostic delay by general diagnostic method, for the 625 subjects with diagnostic delay data and one diagnosis, there was no significant difference in diagnostic delay between general diagnostic method groups (*F*(4) = 0.59, *p* = 0.67; Table S2).

Regarding age at UDN presentation in subjects with one diagnosis, the 684 subjects with one molecular diagnosis (mean = 15.4 ± 16.1 years), compared to the 47 subjects with one nonmolecular diagnosis (mean = 34.4 ± 22.3 years), were significantly younger at presentation (*t*(49.4) = −5.8, *p* < 0.001). Equal variances were not assumed for this test based on Levene’s test for equality of variances.

Regarding age at UDN presentation by number of diagnoses, the 731 subjects with one diagnosis had a mean age of UDN presentation of 16.6 ± 17.2 years (median 10 years, IQR 4–22 years), the 31 subjects with two diagnoses had a mean age of UDN presentation of 17.0 ± 17.0 years (median 11 years, IQR 3–27 years), and the 4 subjects with three diagnoses had a mean age of UDN presentation of 27.5 ± 18.1 years (median 29 years, IQR 9.75–43.75 years). There was no difference in age at UDN presentation between subjects with one and two diagnoses (*t*(760) = −0.12, *p* = 0.45), one and three diagnoses (*t*(733) = −1.26, *p* = 0.10), and two and three diagnoses (*t*(33) = −1.16, *p* = 0.13).

## 4 | Discussion

To our knowledge, the current study is the first to document the proportion of MGD in the UDN. Furthermore, this is the first to report multiple subjects with three concomitant genetic diseases in this cohort, in line with findings of patients with three or even four concomitant genetic diseases (Posey et al. 2017). Of diagnosed UDN subjects, 50/766 (6.5%) had at least one nonmolecular diagnosis, 35/766 (4.6%) had multiple diagnoses (molecular or nonmolecular), 29/766 (3.8%) had multiple Mendelian diagnoses, and 60/766 (8.3%) had at least one molecular non-Mendelian diagnosis; 32/766 (4.2%) had MGD (that is, multiple molecular diagnoses). Given that approximately 30 million individuals in the US are affected by rare diseases (Marwaha, Knowles, and Ashley 2022), a substantial number may have MGD contributing to their disease state and obfuscating their diagnoses. Indeed, multiple subjects with MGD underwent commercial ES, which often came back negative or inconclusive for the subjects’ presenting phenotypes. This indicates that MGD presented a significant diagnostic challenge and that traditional diagnostic methods, including commercially available genome and ES, have room for improvement in the detection of MGD.

The current study’s rate of MGD in the UDN cohort (i.e., 4.2%) was similar to that seen in in previous studies of subjects with confirmed genetic disease (on average, 4.3% in prior studies) (Balci et al. 2017). Still, there exists variation between studies, with rates of MGD ranging from 1.4% to 8.5% (Table 3). One possible reason for this variation may be the differences in the cohorts studied. Studies have differed with regards to the workup required before genetic testing, with many providing no specific details about the prior workup necessary for subject inclusion. Given that the presence of two disease entities could contribute to diagnostic uncertainty, it is possible that a sample of difficult-to-diagnose cases (i.e., those failing prior genetic workup, as seen in some UDN patients) could include a larger number of subjects with MGD than a sample containing any subject sent for genetic workup (including first-time workup). The fact that causative variants were found in only 2 of the 20 pre-UDN exome studies in subjects with MGD suggests that mixed phenotypes and rare variants may contribute to diagnostic difficulty for these subjects. This could account for higher rates of MGD in some studies but not others. Indeed, in subjects with previously assumed phenotypic expansion (i.e., a presumed genetic diagnosis whose presentation does not closely match the typical presentation) attributed to a single variant in known genes, 31.6% were found to have genetic comorbidity (Karaca et al. 2018).

Investigations into the mechanisms behind the incidence of MGD may prove helpful for diagnostics, genetic counseling, and treatment, as has happened with an increased understanding of the inheritance of X-linked and mitochondrial disease (Tinker, Bastarache et al. 2024; Tinker et al. 2021). For example, if MGD is due to the accumulation of de novo mutations for two diseases, clinicians would have to conduct more detailed genetic counseling. Furthermore, clinicians may use family and birth history to determine if a subject has any risk factors for increased rate of MGD (e.g., consanguinity) (Narayanan et al. 2021). To this end, future studies may also investigate if and how the presence of one monogenic variant affects the likelihood of a second variant being inherited or developing de novo and the impact that having two genetic diseases has on fetal viability.

Another interesting finding was that in 3/31 (9.7%) UDN subjects with two diagnoses, the second diagnosis was a non-Mendelian genetic nonmolecular rare disease (Table 1). This presents evidence that having both a rare genetic and non-Mendelian disease can obstruct diagnosis of both genetic and nongenetic disease in the modern genomic era by creating a blended phenotype. Therefore, in addition to insights regarding MGD, the continued work of the UDN may continue to reveal linkages between single-gene changes and polygenic diseases and can prompt clinicians to consider that patients with rare genetic diseases can also have nonmolecular diseases complicating their diagnosis.

Finally, it is also worth noting that subjects with one molecular diagnosis had a significantly earlier age at UDN presentation than those with one nonmolecular diagnosis. While no literature currently exists regarding the exact question of age of diagnosis for molecular versus nonmolecular disease, this result can be interpreted in the context of the presentations of the molecular versus nonmolecular diseases in the current cohort. Molecular diagnoses tended to be those that would be apparent from birth or during childhood development; on the other hand, many of the nonmolecular diagnoses in the current cohort included diseases that would only present later, including age-related dementias and autoimmune diseases. Therefore, by virtue of their lack of a gene-to-phenotype relationship, it makes sense that subjects with nonmolecular diagnoses would present at a later age.

## 5 | Conclusion

Our results give insight into the importance of MGD for medical geneticists. Especially since genetic pleiotropism and comorbidity can contribute to atypical presentations and phenotypes, which may prolong diagnostic odysseys, result in increased patient anxiety and confusion as well as increased healthcare costs. Future research can focus on characterizing the epidemiology of MGD to help elucidate the mechanisms of such disorders as well as guide future diagnostic recommendations for patients with atypical symptom constellations.

## Supplementary Material

Tables S1 to S6

Supplementary Material

Additional supporting information can be found online in the Supporting Information section.

## Figures and Tables

**TABLE 1 |:** Genes and associated diagnoses of subjects with two diagnosed diseases.

ID	Genes	Mendelian diagnoses	Inheritance	Variant	Diagnostic method	Non-mendelian diagnoses	Diagnostic method
1	*COL6A*	Bethlem myopathy 1	AD	NM_001848.2:c.930+189C>T(null)	Reanalysis of genome-scale data	—	—
	*NSD2*	*NSD-2*-related condition	AD	NM_133330.2:c.957G>T(p.Arg319Ser)	Reanalysis of genome-scale data		
2	*PRELP*	Osteosclerosis	AD	NM_002725.3:c.778G>A(p.Gly260Arg)	Genome-scale sequencing	—	—
	*PMP22*	Charcot–Marie–Tooth Disease, Type 1A	AD	Duplication(17:14087933–15500645)	Microarray testing		
3	*PTCH1*	Gorlin syndrome	AD	NM_001083602.2:c.3+314G>T(null)	Reanalysis of genome-scale data	—	—
	*CHD7*	CHARGE syndrome	AD	NM_017780.3:c.8077G>C(p.G2693R)	Reanalysis of genome-scale data		
4	*ENG*	Hereditary hemorrhagic telangiectasia, type 1	AD	NM_001114753.3:c.816+6T(null)	Reanalysis of genome-scale data	Systemic lupus erythematosus	Multi- specialist evaluation
5	*CITED2*	Atrial septal defect 8	AD	NM_006079.4:c.701A>C(p.Glu234Ala)	Genome-scale sequencing	—	—
	*CHD2*	*CHD2*-related neurodevelopmental disorder	AD	NM_001271:c.−367G>C(null)	Re-analysis of genome-scale data		
6	*PMP22*	Hereditary neuropathy with liability to pressure palsies	AD	NM_000304:c.353C>T(p.T118M)	Reanalysis of prior exome data	—	—
	*QRICH1*	Ververi-Brady syndrome (NS, NS)	AD	NM_017730:c.1378C>T(p.Q460X)	Reanalysis of prior exome data		
7	*NRCAM*	*NRCAM*-related neurodevelopmental disorder	AR	NM_001193582.1:c.230+824G>A(null) & NM_001193582:c.164A>G (p.Asp55Gly)	Genome-scale sequencing	—	—
	*KRAS*	Mosaic *KRAS*-related RASopathy (NS, NS)	AD	NM_001193582.1:c.164A>G(p.D55G)	Genome-scale sequencing		
8	*ALPL*	Adult hypophosphatasia	AD	NM_000478.6:c.673T>C(p.Tyr225His)	Genome-scale sequencing	—	—
	*CYP27A1*	*CYP27A1*-associated osteoporosis	AD	NM_000784:c.1183C>T(p.Arg395Cys)	Reanalysis of genome-scale data		
9	*PPP5C*	*PPP5C*-related condition	AD	NM_006247.3:c.139G>A(p.Ala47Thr)	Reanalysis of prior clinical exome data	—	—
	*VARS1*	Neurodevelopmental disorder with microcephaly, seizures, and cortical atrophy	AR	NM_006295.2:c.3288G>T(p.Glu1096Asp & NM_006295.2:c.2590A>T;2592C>A(p. S864X)	RNAseq		
10	*CREBBP*	Rubinstein-Taybi syndrome 1	AD	NM_004380:c.3699–1469+1579del(null)	Optical genome mapping	—	—
	*FMR1*	Fragile X syndrome	AD	Pathogenic repeat expansion	Repeat-primed PCR		
11	*WNT10A*	Selective tooth agenesis 4	AD	NM_025216.2:c.682T>A(p.Phe228Ile)	Genome-scale sequencing	—	—
	*SOX4*	Coffin-Siris syndrome 10	AD	NM_003107.2:c.1040C>A(p.S347*)	Genome-scale sequencing		
12	*FBN1*	Marfan syndrome	AD	NM_000138.5:c.4786C>T(p.Arg1596*)	Genome-scale sequencing	—	—
	*TRPS1*	Trichorhinophalangeal syndrome	AD	NM_014112.5:c.1630C>T(p.Arg544*)	Genome-scale sequencing		
13	*SCN4A*	Hypokalemic periodic paralysis	AD	NM_000334.4:c.3403C>A(p.R1135S)	Genome-scale sequencing	—	—
	*POC1A*	SOFT syndrome	AR	NM_015426.5:c.79_87delTTCAGTATC(p. Phe27_Ile29del)Homozygous	Genome-scale sequencing		
14	*ATP1A3*	*ATP1A3*-related disorder	AD	NM_152296:c.2767G>A(p.D923N)	Reanalysis of exome-scale data	—	—
	*TSPEAR*	*TSPEAR*-related disorder of tooth and hair follicle morphogenesis	AR	NM_144991:c.1726_1728delinsAA(p. V576fs) & NM_144991:c.589C>T(p. R197X)	Reanalysis of exome-scale data		
15	*NSD2*	*WHSC1* (*NSD2*)-related condition	AD	NM_133330.2:c.2519G>T(p.Gly840Val)	Genome-scale sequencing	—	—
	*NEUROD2*	*NEUROD2* neurodevelopmental syndrome	AD	NM_006160.4:c.488T>C(p.Leu163Pro)	Reanalysis of genome-scale data		
16	—	Chromosome 1q21.1 duplication syndrome	N/a	—	Microarray	Multiple sclerosis	Multi- specialist evaluation
17	*TNFRSF13B*	Common variable immunodeficiency 2	AD	NM_012452.2:c.823C>T(p.P275S)	Known prior to enrollment in UDN	—	—
	*TPSAB1*	Hereditary alpha-tryptasemia syndrome	AD	NM_182916.2:c.1081C>T(p.R361C)	Copy number analysis		
18	*GNB1*	GNB1 encephalopathy	AD	NM_002074.4:c.716A>G(p.N239S)	Genome-scale sequencing	—	—
	*MT-TK*	Myoclonic epilepsy with ragged-red fibers	—	NC_012920.1:m.8344A>G(null) identified on mt DNA sequencing	Mitochondrial genome sequencing		
19	*PCDHA@*	Protocadherin-alpha-related cerebellar atrophy	NA	Disruption by translocation	Genome-scale sequencing	—	—
	*BRAF*	*BRAF* disruption	NA	seq[GRCh38] t(5; 7)(q31.3; q34)	Genome-scale sequencing		
20	*NRXN1*	*NRXN1*-related Pitt-Hopkins- like syndrome 2	AR	NM_001135659:c.1412G>A(p.Ser471Asn) & NM_004801.5:c.3364+13144G>A(null)	Reanalysis of prior exome data	—	—
	*PHACTR1*	Novel *PHACTR1*-related neurodevelopmental disorder	AD	NM_030948.3:c.251–63908_415+29843dup(duplication of exon 5)	Genome-scale sequencing		
21	*BAP1*	Kury-Isidor syndrome	AD	NM_004656.3:c.271T>C(p.C91R)	Reanalysis of prior clinical exome data	—	—
	*CTNNA1*	*CTNNA1*-associated familial exudative vitreoretinopathy	AD	NM_001903.5:c.2191C>T(p.Arg731*)	Reanalysis of prior clinical exome data		
22	*SOX11*	Coffin-Siris syndrome	AD	NM_003108.3:c.292delT(p. Phe98SerfsTer108)	Genome-scale sequencing	—	—
	*GJB2*	Autosomal recessive deafness type 1A	AD	NM_004004.5:c.269T>C(p.Leu990Pro)	Genome-scale sequencing		
23	*POLG*	Mitocondrial DNA depletion syndrome, NOS	AR	NM_002693.2:c.641C>T(p.Ala214Val) & NM_002693.2:c.391T>C(p.Tyr131His)	Genome-scale sequencing	Primary progressive multiple sclerosis	Clinical diagnosis
24	*COL5A2*	Ehlers-Danlos syndrome, classic type	AD	NM_000393:Exons 8–49 Deletion(null)	Concordance array	—	—
	*IRAK4*	Immunodeficiency due to interleukin-1 receptor-associated kinase-4 deficiency	AR	NM_016123.3:c.364C>T(p.Q122*) & heterozygous 12:43775405–43788500 deletion	Genome-scale sequencing		
25	*SPATA5L1*	Neurodevelopmental disorder with hearing loss and spasticity	AR	c.527G>T (p.G176V) & c.95G>A (p.G32D)	Reanalysis of prior whole genome sequencing	—	—
	*UMPS*	Orotic aciduria (NS, NS)	AR	c.857T>A (with raised ortic acid)—has biochemical features of the disorder so diagnosed by UDN clinical team	Panel testing		
26	*MPO*	Myeloperoxidase deficiency	AR	NM_000250.2:c.1705C>T(p.Arg569Trp) & NM_000250.2:c.2031–2A>C(null)	Whole exome sequence	—	—
	*MCM6*	*MCM6*-related neurodevelopmental disorder	AD	NM_005915.5:c.715G>A(p.Gly239Ser)	Reanalysis of genome-scale data		
27	*FCSK*	Congenital disorder of glycosylation with defective fucosylation 2	AR	NM_145059.2:c.667T>C (p.S223P), NM_145059.2:c.2047C>T(p.R683C)	Reanalysis of exome-scale data	—	—
	*HCFC1*	Methylmalonic aciduria and homocystinuria, cblX type	AD	NM_005334.3:c.344C>T(p. A115V)—Do novo variant	Genome-scale sequencing		
28	*CFTR*	Cystic fibrosis	AR	NM_000492:c.3909C>G(p. N1303K) (Homozygous)	Known prior to enrollment	—	—
	*AP4M1*	Spastic paraplegia 50	AR	NM_004722:c.929+5G>A(null) (Homozygous)	Reanalysis of exome-scale data		
29	*WWOX*	Early infantile epileptic encephalopathy 28	AR	NM_016373.3:c.689A>C(p.Q230P) & NM_016373.3:exon 5 deletion(null)	Genome-scale sequencing	—	—
	*GRIA4*	Neurodevelopmental disorder with or without seizures and gait abnormalities	AD	NM_000829.3:c.247+15999T>G(null)	Genome-scale sequencing		
30	*FAT4*	van Maldergem syndrome 2	AR	NM_001291303:c.10560G>A(p. Met3520Ile) & NM_001291303:c.8021A>T(p. Asp2674Val)	Genome-scale sequencing	Diploid/triploid mosaicism	Found on CMA
31	*ALPL*	Hypophosphatasia	AD	NM_000478.6:c.1364G>A(p.Gly455Asp)	Genome-scale sequencing	—	—
	*SLC9A3R1*	Hypophosphatemic nephrolithiasis/ osteoporosis 2	AD	NM_004252.5:c.458G>A(p.Arg153Gln)	Reanalysis of prior genome data		

**TABLE 2 |:** Genes and associated diagnoses of subjects with three diagnosed diseases.

ID	Genes	Mendelian diagnoses	Inheritance	Variants	Diagnostic method
32	*FLG*	Susceptibility to atopic dermatitis 2	AD	NM_002016.1:c.2476C>T(p.R826*)	Genome-scale sequencing
	*FLCN*	Birt-Hogg-Dube syndrome 1	AD	NM_144997.5:c.1285dupC(p.H429Pfs* 27)	Genome-scale sequencing
	*SATB1*	*SATB1*-related neurodevelopmental disorder	AD	NM_002971.6:c.740_741delAA(p.K247Rfs*5)	Genome-scale sequencing
33	*POC5*	Scoliosis	AD	NM_001099271:c.1363G>C(p.Ala455Pro)	Genome-scale sequencing
	*MSL2*	*MSL2*-associated autism spectrum disorder	AD	NM_018133:c.694_697del(p.Ser232Tfs*10)	Genome-scale sequencing
	*NOTCH1*	Aortic valve disease	AD	NM_017617:c.2812C>T(p.Arg938Trp)	Genome-scale sequencing
34	*MME*	Charcot–Marie–Tooth disease, axonal, type 2 T	AD	NM_000902.3:c.202C>T(p.Arg68Ter)	Genome-scale sequencing
	*SLC38A8*	Foveal hypoplasia 2, with or without optic nerve misrouting and/or anterior segment dysgenesis	AR	NM_001080442.2:c.848A>C(p.Asp283Ala) & NM_001080442.2:c.913T>C(p.Ser305Pro)	Genome-scale sequencing
	*F5*	Thrombophilia due to activated protein C resistance	AD	NM_000130.4:c.1601G>A(p.Arg534Gln)	Directed clinical testing based on phenotype
35	*SERPINA1*	Alpha-1-antitrypsin deficiency (*Zz*)	AD	NM_001127701.1:c.1096G>A(p.E366K)	Genome-scale sequencing
	*COL4A1*	HANAC	AD	NM_001845.5:c.4357C>T(p.Q1453*)	Genome-scale sequencing
	Alignment *BARD1*	Breast cancer susceptibility	AD	NM_000465.3:c.860_861delAG(p.E287Vfs*5)	Genome-scale sequencing

**TABLE 3 |:** Studies examining rates of multiple genetic disease in undiagnosed cohorts.

Study	Setting	Prior diagnostic testing?	Proportion of solved cases with MGD
Yang et al. (2013)	Laboratory, referred by clinician	Not specified	6.5% (4/62)
Yang et al. (2014)	Laboratory, referred by clinician	No	4.6% (23/504)
Lee et al. (2014)	Laboratory, referred from academic center	No	1.4% (3/213)
Farwell et al. (2015)	Laboratory, referred by clinicians	Not specified	7.2% (11/152)
Yavarna et al. (2015)	Medical genetics clinic, referred by a clinician	Not required, but subjects “typically” had prior workup	6.7% (6/89)
Retterer et al. (2016)	Laboratory, referred by a clinician	Not specified	3.1% (27/876)
Posey et al. (2016)	Laboratory, referred by a clinician	Not specified	7.1% (6/85)
Posey et al. (2017)	Laboratory, referred by a clinician	Not specified	4.9% (101/2076)
Balci et al. (2017)	Clinical consortium, standard diagnostic workup failed (Beaulieu et al. 2014)	No, but “vast majority” of subjects had already experienced diagnostic odyssey	3.5% (8/2206)
Smith et al. (2019)	Laboratory, referred by a clinician	Not specified	8.5% (153/1792)
Narayanan et al. (2021)	Clinic, referral means not specified	Not specified	2.5% (13/528)
Gimeno et al. (2024)	Clinical consortium, standard diagnostic workup failed	Not required, but done for many subjects	4.2% (32/766)

## Data Availability

The data that support the findings of this study are available from the corresponding author upon reasonable request.
