# Supplementary material for: Prevalence of Individuals With Multiple Diagnosed Genetic Diseases in the Undiagnosed Diseases Network
Source: Am J Med Genet A. Author manuscript; Available in PMC 2025 Jul 8. (PMC12234204; doi:10.1002/ajmg.a.63888)
Supplement: Tables S1 to S6 [file NIHMS2085995-supplement-Tables_S1_to_S6.docx]

Table S1: Racial demographics of the diagnosed UDN cohort

| Race | Frequency (percent) |
| --- | --- |
| White | 608 (79.4%) |
| Asian | 61 (8.0%) |
| Black or African American | 39 (5.1%) |
| Mixed Race | 36 (4.7%) |
| Unspecified | 11 (1.4%) |
| Other | 6 (0.8%) |
| American Indian or Alaskan Native | 4 (0.5%) |
| Native Hawaiian or Other Pacific Islander | 1 (0.1%) |

Table S2: Diagnostic delay by general diagnosis method for patients with one diagnosis.

| Diagnostic Method | N | Diagnostic Delay (years; mean ± SD) |
| --- | --- | --- |
| Genome-scale sequencing | 505 | 10.8 ± 9.3 |
| Directed clinical testing based on phenotype | 55 | 9.2 ± 6.9 |
| Diagnosis made primarily based on clinical grounds | 38 | 9.9 ± 10.0 |
| Non-sequencing genome-wide diagnostic array | 20 | 12.0 ± 12.4 |
| Other | 7 | 12.3 ± 5.3 |
| Total | 625 | 10.7 ± 9.2 |

Table S3: Diagnostic delay by number of diagnoses for patients with one molecular diagnosis, one non-molecular diagnosis, one of any type of diagnosis, two diagnoses, and three diagnoses.

| Group | N | Diagnostic Delay (years; mean ± SD) |
| --- | --- | --- |
| One molecular diagnosis |  |  |
| One non-molecular diagnosis |  |  |
| One diagnosis (any) |  |  |
| Two diagnoses |  |  |
| Three Diagnoses |  |  |

Table S3: Specific diagnostic methods for all diagnoses made with genome-scale sequencing

| Type | Frequency |
| --- | --- |
| Reanalysis of prior data | 113 |
| Baylor - pathogenic | 96 |
| Baylor - likely pathogenic | 37 |
| Clinical site analysis of Baylor exome data | 32 |
| Clinical site analysis of HudsonAlpha genome data | 30 |
| Baylor - VUS | 26 |
| Known prior to enrollment | 14 |
| HudsonAlpha - pathogenic | 13 |
| Baylor - research finding | 13 |
| HudsonAlpha - research finding | 7 |
| HudsonAlpha - VUS | 7 |
| HudsonAlpha - likely pathogenic | 5 |
| Baylor - exome data | 5 |
| Clinical site analysis of Baylor genome data | 5 |
| Unspecified | 2 |
| Panel | 1 |
| GeneDx Genomics | 1 |
| WES | 1 |
| Other clinical genome sequencing at GeneDx | 1 |
| RNAseq with whole genome seq | 1 |
| Expansion repeat HTT expansion study | 1 |
| Pathology | 1 |
| RNAseq | 1 |

NB: “Baylor” or “HudsonAlpha” refers to genome- or exome-wide testing performed at a given laboratory.

Table S4: Specific diagnostic methods for all diagnoses made with phenotype-based clinical testing

| Type | Freq |
| --- | --- |
| Single gene testing | 21 |
| Labs | 10 |
| Panel testing | 9 |
| Imaging | 5 |
| Pathology | 5 |
| Unspecified | 3 |
| mtDNA sequencing | 3 |
| Methylation analysis | 2 |
| Long-term sequencing | 1 |
| Karyotype | 1 |
| WES | 1 |
| Pathogenic exome sequencing | 1 |
| Pathogenic exome or genome sequencing | 1 |

Table S5: Specific diagnostic methods for all diagnoses made primarily on clinical grounds

| Type | Freq |
| --- | --- |
| Diagnosis of Exclusion | 4 |
| Pathology | 3 |
| Imaging only | 2 |
| Labs | 2 |
| Labs and imaging | 1 |
| Labs and history | 1 |
| Verified with genetics based on history | 1 |

Table S6: Specific diagnostic methods for non-sequencing genome-wide diagnostic arrays

| Type | Frequency |
| --- | --- |
| Microarray | 13 |
| Karyotype | 3 |
| Known prior to enrollment | 3 |
| Optical genome mapping | 2 |
| Microarray detected by concordance array run with exome | 1 |
| Microarray and karyotype | 1 |
| Fragile X repeat analysis | 1 |
| SNP analysis | 1 |
| Methylation testing | 1 |
| Copy number analysis | 1 |
| Combination of exome sequencing, RNAseq, clinical correlation, targeted Sanger sequencing | 1 |

Table S7: Specific diagnostic methods for all other methods of diagnosis

| Type | Frequency |
| --- | --- |
| RNAseq | 2 |
| Reanalysis of prior data | 1 |
| Model organism functional studies | 1 |
| WES | 1 |
| Cell-based analysis | 1 |
| RNAseq and genome search | 1 |
| Karyotyping, FIRSH, long-read genome, methylome, epigenome, and transcriptome sequencing | 1 |
| Transcriptome and genome data | 1 |
| Exome sequencing and targeted biochemical testing | 1 |
| Karyotyping and RNAseq | 1 |
| Long-read sequencing | 1 |
| RNAseq and sequencing | 1 |
| WGS | 1 |
| RNAseq and WES | 1 |
| Known prior to enrollment | 1 |
| RNAseq and targeted deep sequencing | 1 |
| RNAseq and reanalysis of pror data | 1 |
| Newborn Screen | 1 |
